# Supplementary material for: Trial-history biases in evidence accumulation can give rise to apparent lapses in decision-making
Source: Nat Commun. 2024 Jan 22;15:662. doi: 10.1038/s41467-024-44880-5 (PMC10803295; doi:10.1038/s41467-024-44880-5)
Supplement: Supplementary file 1 — Supplementary Information [file 41467_2024_44880_MOESM1_ESM.pdf]

Supplementary Information

Trial-history biases in evidence accumulation can give rise to apparent lapses in decision-making

Diksha Gupta, Brian DePasquale, Charles D. Kopec, Carlos D. Brody

List of Figures

1 Exponential filtering for initial state setting approximates Bayesian prior updates under assumptions of non-stationarity 2
2 Influence of within- and across-trial parameters on history-dependent modulation of biases and lapses in the psychometric function. 3
3 Performance measures across the rat dataset 4
4 Logistic fits to psychometric curves reliably recover performance asymptotes i.e lapse rates 5
5 Two variants of the accumulator with HISt model with different kinds of true lapses perform equally well 6
6 An accumulator model with history modulation of just true lapses performs much worse. 8
7 Interpreting initial state updates through the lens of statistical inference 9
8 Fits of the accumulator model with history-modulated initial states (and additional true lapses arising from motor error) to choices and reaction times of individual rats 11
9 Accumulator model with HISt and true lapse variants fit to the RT dataset 12

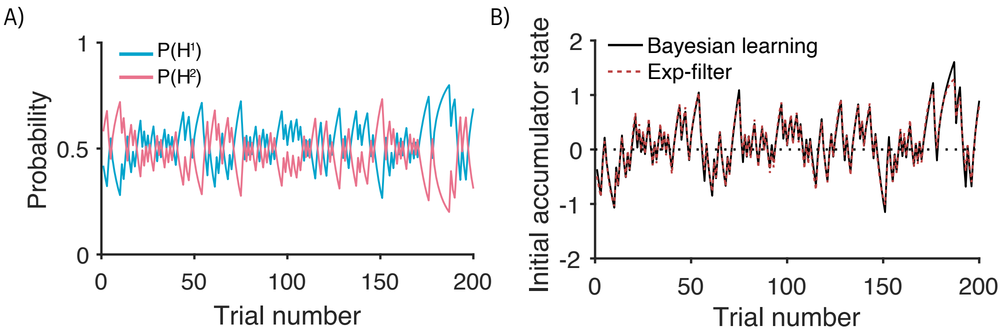

Supplementary Figure 1: **Exponential filtering for initial state setting approximates Bayesian prior updates under assumptions of non-stationarity A**: Example of a mis-belief in a non-stationary prior. Traces represent belief about prior probability of two hypotheses H^1 and H^2 inferred from a random sequence of trials drawn from a stationary symmetric prior, under the misbelief that the prior is occasionally undergoing unsignalled jumps. Such an assumed generative model is often referred to as the Dynamic Belief Model (DBM; Yu and Cohen[1]). **B**: Initial state updates corresponding exactly to the fluctuating prior beliefs in (A) that emerge from Bayesian learning (black line), plotted against approximate initial states derived from exponential filtering (dotted red line) of past choices and outcomes. The exponential filter provides a good approximation of exact Bayesian updates, while being more expressive and flexible to capture the possibility of other generative models and corresponding update rules.

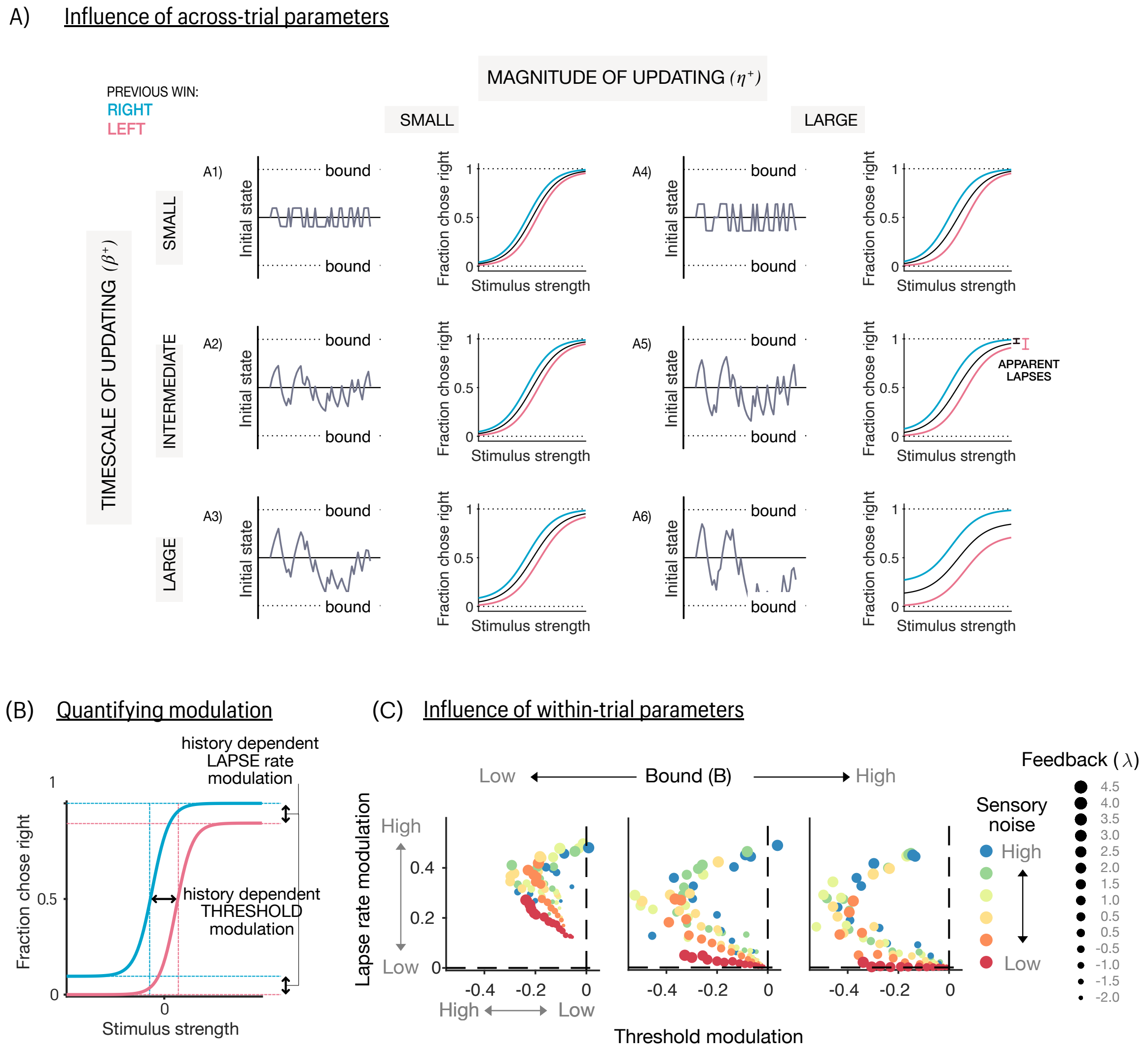

Supplementary Figure 2: **Influence of within- and across-trial parameters on history-dependent modulation of biases and lapses in the psychometric function. (A)** Influence of across-trial parameters on history-dependent modulation: Effects of varying magnitude of trial-by-trial updating (η - columns) and timescale of updating (β - rows) on initial state trajectories (gray lines) and psychometric curves (black - conditioned on previous trial being a win, blue - previous trial right win, pink - previous trial left win). (Top row) Small timescales ($\beta = 0.01$) of updating lead to fast fluctuations in initial states, and mostly horizontal shifts in psychometric curves with trial history, for both small ($\eta = 0.14$) and large ($\eta = 0.2$) magnitudes of updating (A1 and A4). (Middle row) Intermediate timescales of updating ($\beta = 0.65$) lead to slower fluctuations in initial state that have a cumulative effect across trials. For large magnitudes of updating (A5) these can give rise to apparent lapses (black intervals) as well as history-dependent modulation of these lapses (pink intervals). (Bottom row) Long timescales of updating ($\beta = 0.84$) lead to stronger cumulative initial state biases across trials, yielding apparent lapses and lapse rate modulations even for small magnitudes (A3). When combined with large magnitudes of updating (A6) lead to initial states that sometimes exceed the bounds, leading to a combination of apparent lapses (initial states within bounds) and deterministic, stimulus-independent decisions (initial states outside bounds). Caption continued on next page.

Supplementary Figure 2: (Previous page.) It is important to note that, apart from the final case, the apparent lapses stem from the fact that diverse initial states lead to psychometric curves characterized by varying "sensitivities", resulting in psychometric curves with heavy tails. When these curves are approximated using the logistic function, the heavy tails are accounted for as lapse rates. If measurements were conducted for stimulus strengths that are even higher, the inherent heaviness of the psychometric curve's tails would become evident, rendering logistic functions inadequate for providing a suitable fit. Measures of overdispersion (e.g. Schütt et al.[2]) can be used to detect such heavy-tailedness, when measuring choices at higher and higher stimulus strengths is not feasible. **(B)** Quantifying modulation of psychometric properties: The difference between psychometric curves conditioned on previous wins (blue) or losses (pink) can be quantified using two metrics - the horizontal distance between the midpoints of psychometric curves ("threshold modulation") and the vertical distance between its asymptotes ("lapse rate modulation") **(C)** Effects of varying the parameters of the within-trial drift diffusion model (DDM) on history-dependent threshold (x-axis) and lapse rate modulations (y-axis). Colors denote levels of sensory noise, size of dots denote values of the feedback parameter of the DDM. The feedback parameter determines if the accumulation is leaky ($\lambda < 0$, ignores early evidence), perfect ($\lambda = 0$, uses all evidence) or impulsive ($\lambda > 0$, ignores later evidence). Plots from left to right are for low, intermediate and high values of bound respectively. High bounds predominantly give rise to threshold modulations, however high positive values of feedback and higher levels of sensory noise additionally produce lapse rate modulations. Lapse rate modulations are dramatically increased by lower bounds for many different values of feedback and by higher values of sensory noise.

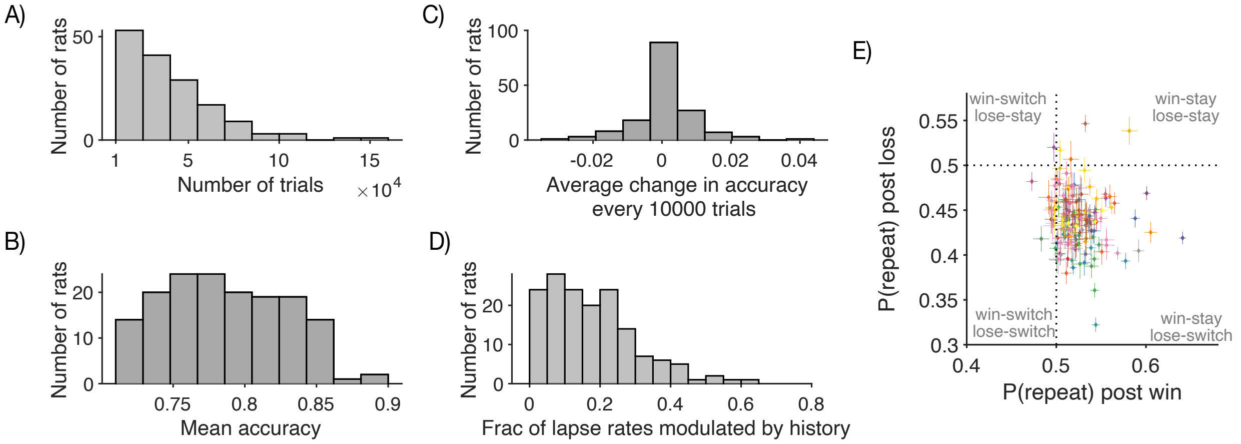

Supplementary Figure 3: **Performance measures across the rat dataset (A)** Histogram of trial counts for all rats (n = 152) in the population. Most rats completed on the order of $1e^4$ trials. **(B)** Histogram of mean accuracy showing that rats showed good performance on the task (mean accuracy $\pm$ SD: 0.79 ± 0.04). **(C)** Average change in mean accuracy every 10000 trials, showing that rats' performance was stable over time, reflecting asymptotic behavior rather than task acquisition. **(D)** Histogram of history-modulated lapse rates as a fraction of total lapse rates. A sizeable portion of the population had non-zero fractions, suggesting that history-dependence could potentially account for substantial lapse rate variance. **(E)** Scatter comparing repetition bias following wins and losses. Each point is a rat, error bars are Wilson binomial CIs. Most of the population occupied the bottom right quadrant, showing a "win-stay, lose-switch" bias i.e. repetitions following wins and alternations following losses.

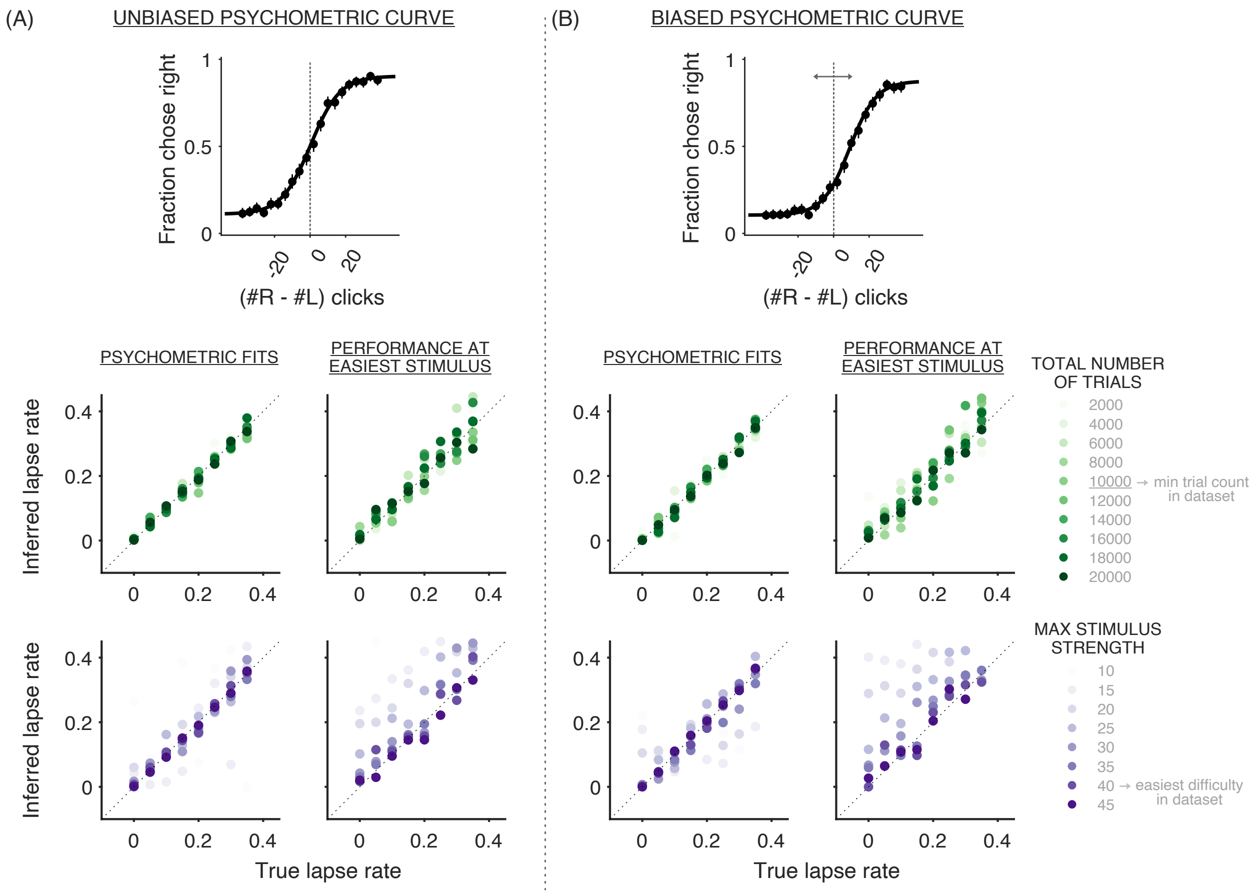

Supplementary Figure 4: **Logistic fits to psychometric curves reliably recover performance asymptotes i.e lapse rates** For this study, it is crucial to reliably estimate the asymptotes of psychometric curves in order to measure lapses. We confirmed that the logistic fits (see Methods, Psychometric curves) consistently yield reliable recovery of lapse rates across various conditions. These conditions include diverse total trial counts (depicted in middle panels **A** and **B**) and variations in the maximum stimulus strength at which choice behavior is measured (illustrated in bottom panels **A** and **B**). We find that the method accurately estimates the true lapse rates in scenarios when choices are biased (**A**) as well as unbiased (**B**). We contrast the performance of this method with an alternative approach of estimating asymptotes by simply measuring the performance at easiest stimuli (i.e. maximum stimulus strengths) e.g. as in Wang et al.[3]. This approach yields biased estimates particularly when the trial counts are low or when choices are measured only at low stimulus strengths.

Across all simulations, the sensitivity of psychometric curves was set to 0.125, similar to values typically seen in our dataset (Fig 2C). For simulations involving varying trial counts (middle panels), the maximum stimulus strength was set at 40. Simulations exploring distinct maximum stimulus strengths (bottom panels) were carried out with 10,000 simulated choices. The top panels represent choices simulated with true lapse probability of 0.2. Each point corresponds to estimates derived from individual simulated datasets, error bars have been omitted for clarity of visualization.

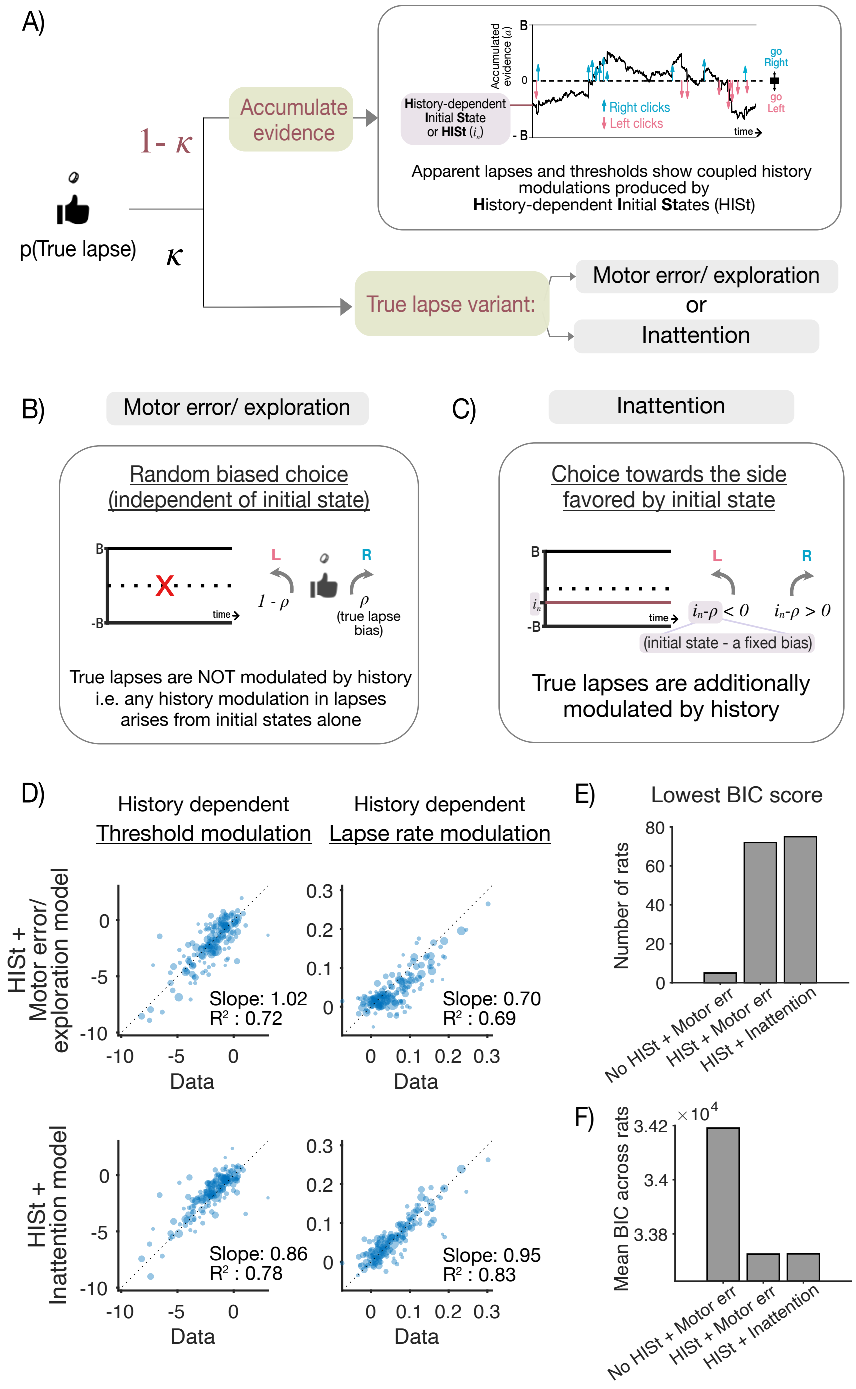

Supplementary Figure 5: **Two variants of the accumulator with HISt model with different kinds of true lapses perform equally well (A)** Schematic of accumulator with HISt (top), which produces apparent lapses and thresholds that are both modulated by history, with two variants of true lapses (bottom) - those due to motor errors/exploration, and those due to inattention (Figure adapted with permission from Bingni W. Brunton et al., Rats and Humans Can Optimally Accumulate Evidence for Decision Making. Science 340,95-98(2013). DOI:10.1126/science.1233912) **(B)** Motor error/exploration variant, that occasionally chooses a random action with some bias (ρ) irrespective of the initial state, reflecting an error in motor execution or random exploration. This model produces true lapses that are not modulated by history, such that any history modulations arise from HISt alone. Caption continued on next page.

Supplementary Figure 5: (Previous page.) **(C)** Inattentional variant, that occasionally fails to attend to the stimulus, and relies on the initial state to make an informed, deterministic decision based on the difference between the initial state and a bias (ρ). In this model, true lapses are also modulated by history in addition to apparent lapses and thresholds. **(D)** Individual differences in history effects captured by different models: History modulations of threshold (left) and lapse rate (right) parameters measured from psychometric fits to the raw data (x-axis) v.s. model predictions (y-axis). (Top): Motor error/exploration model has no history dependence in true lapses, yet captures a majority of the variance in both threshold and lapse rate modulations [$R^2 = 0.72$ (threshold parameter), $R^2 = 0.69$ (lapse rate parameter)], and shows good correspondence with both parameters, as evidenced by the points lying close to the unity line [slope$= 1.02$ (threshold parameter), slope $= 0.70$ (lapse rate parameter)]. This suggests that these modulations can be captured by the comodulations in apparent lapses and thresholds produced by HISt. (Bottom): same as Top but for Inattention model. The inattention model allows true lapses to additionally depend on history, and captures slightly more variance in history modulations [$R^2 = 0.78$ (threshold parameter), $R^2 = 0.83$ (lapse rate parameter)]. However, it does so at the expense of correspondence with thresholds [slope$= 0.86$ (threshold parameter), slope $= 0.95$ (lapse rate parameter)]. This marginal improvement suggests that comodulations in thresholds and lapse rates largely reflect apparent lapses arising from HISt, rather than true lapses of either kind. **(E)** Distribution of best fitting model variants for individual rats: Overall bar height for each variant denotes the total number of rats for which that variant scored the lowest BIC score. Inattention variant won in marginally more rats than motor error (inattention: 75/152 rats, motor error: 72/152 rats). **(F)** Population model comparison using mean BIC score (n = 152) across rats. Lower scores indicate better fits. Scores are comparable across variants, marginally favoring motor-error over inattention (Mean BIC score for motor error/exploration: 33725.64, inattention: 33726.25).

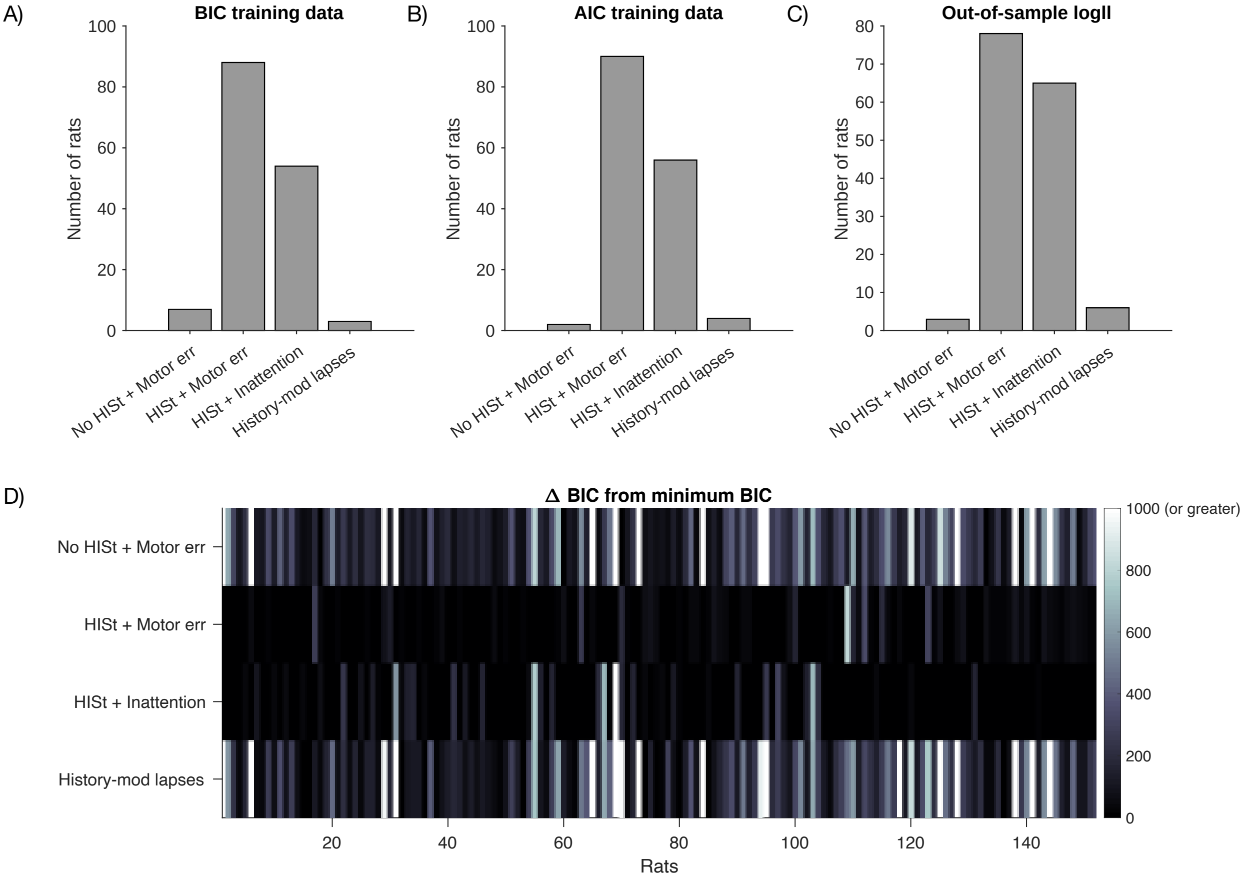

Supplementary Figure 6: **An accumulator model with history modulation of just true lapses performs much worse.** To demonstrate that the trial-history effects on the initial state of the accumulator are distinguishable from trial-history effects on true lapses, we compared the HISt models with a model variant in which trial-history exclusively modulated lapses (History-mod lapses). This is important since the BIC scores were similar for model variants with history modulation of true lapses (HISt + Inattention, Supp Fig. 5). We found that this model performs much worse as measured by BIC **(A)**, AIC **(B)** and Out-of-sample loglikelihood (20% of choices) **(C)**. These plots show the distribution of rats that are best fit by these model variants based on these individual metrics. **(D)** Difference in BIC scores from the model variant with best score are shown for each individual rat. These results provide strong evidence that history modulation of the initial state plays a crucial role in achieving better model fits and effectively capturing the comodulation of threshold and lapse rate parameters. Conversely, the true lapse modulations make only a minor contribution the overall performance.

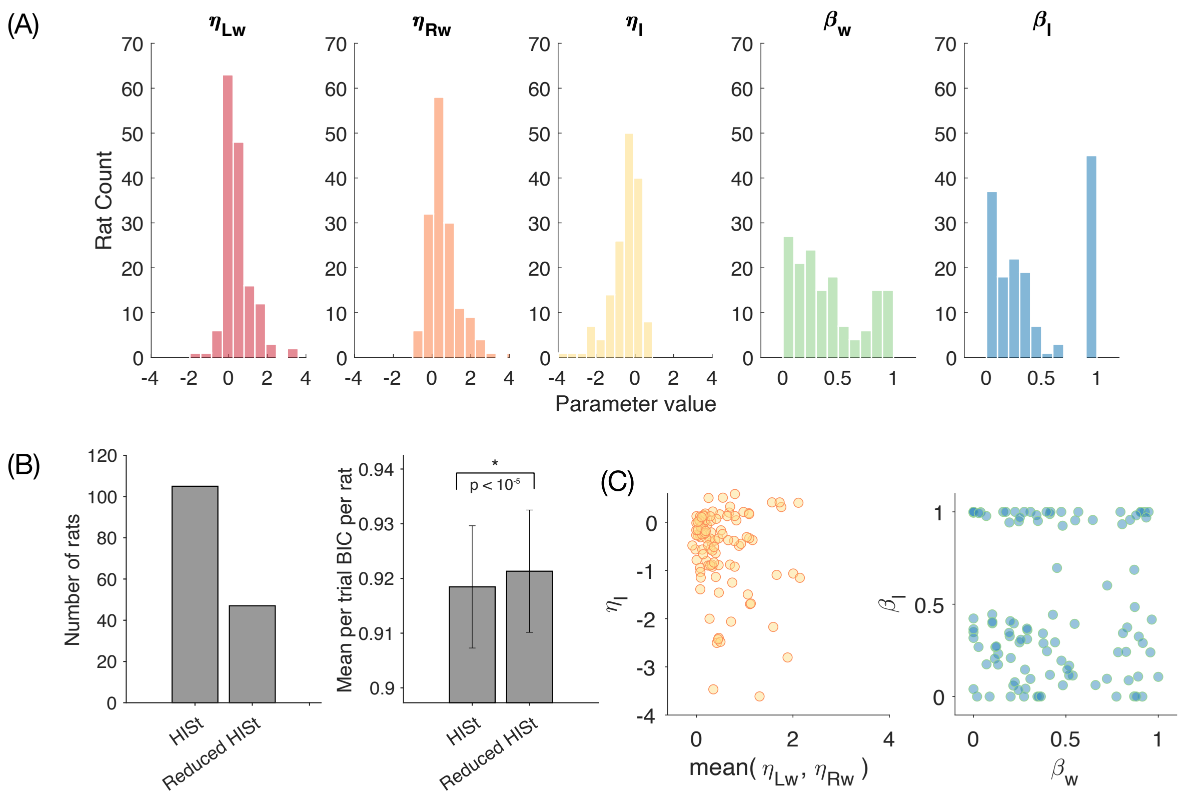

Supplementary Figure 7: **Interpreting initial state updates through the lens of statistical inference** (**A**) Distribution of initial state updating parameters across the population of rats. Median magnitudes of updating following wins (first two panels) and losses (third panel) were not significantly different in their absolute value (median: mean(η_{Lw}, η_{Rw}) $= 0.36$, $\eta_l = -0.35$, $p = 0.11$, one-sided Wilcoxon signed-rank test) however they did differ in their signs - wins tended to induce a tendency to repeat (positive X values) while losses induced a tendency to switch (negative X values). Median timescales of updating following wins (0.34) and losses (0.29; fourth, fifth panels) were not significantly different ($p = 0.46$, Wilcoxon signed-rank test). (**B**) Comparison of the "HISt" model with a reduced version that approximates optimal statistical inference in the Dynamic Belief Model (Supp Fig 1, Yu and Cohen[1]). The optimal update rule for the DBM corresponds to a restricted regime of the HISt model, where the magnitude and timescale parameters are set to be the same for all trial types i.e. left-win, right-win, left-loss and right-loss. We compared the fits of this "Reduced HISt" model with the HISt model, and found that it was not supported by BIC in the majority of rats (Left panel: overall bar height denotes the total number of rats for which that model variant scored the lowest BIC score, HISt was winning model in 105/152 rats). Moreover, the unconstrained HISt model performed better on average per trial (Right panel: Model comparison using BIC by pooling per trial BIC score across rats and computing mean. Lower scores indicate better fits. Mean per trial BIC scores across rats were significantly lower for model with HISt, $p = 2.23 \times 10^{-6}$, paired t-test. Error bars are SEM). Overall, this means that while some rats' (47/152) updating strategy was consistent with optimal inference in a DBM, most rats' parameters did not occupy this restricted regime. (**C**) Joint distribution of updating magnitude (left) and timescale (right) parameters following wins and losses. Caption continued on next page.

Supplementary Figure 7: (Previous page.) Contrary to the expectation from optimal updating in a DBM, the population of rats did not show a significant correlation between these parameters following wins and losses (magnitude of updating: $r = -0.12, p = 0.22$, timescale: $r = 0.02, p = 0.78$). However, the subset of 47 rats best fit by the Restriced HISt model (not shown) did show a significant correlation in the magnitude parameter alone ($r = -0.45, p = 0.001$). These findings could be potentially consistent with a statistical model in which wins and losses are treated differently for updating (e.g. Hermoso-Mendizabal et al.[4], Karlsson et al.[5]). This could be due to the differential information signalled by wins and losses - while both are relevant for estimation of current prior, losses might additionally signify an abrupt change in the environment warranting a “re-drawing” of prior estimates. While such a model bears resemblance to DBM, its precise characteristics are yet to be fully defined.

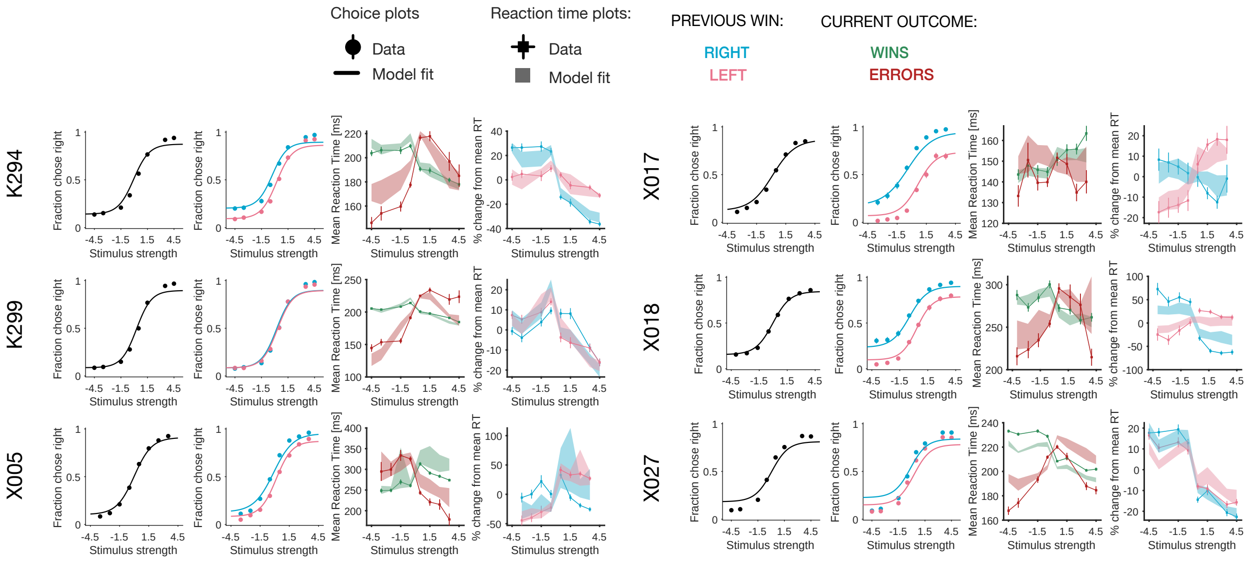

Supplementary Figure 8: **Fits of the accumulator model with history-modulated initial states (and additional true lapses arising from motor error) to choices and reaction times of individual rats**. Each horizontal set of 4 panels shows fits to an individual rat, and each of the 4 columns depicts a different behavioral measure summarizing choices (first column, data points: mean fraction rightward choices ± binomical CIs, psychometric curve), reaction times (third column, win RTs in green and error RTs in red; mean ± SEM), and history modulations in choices/reaction time (second/fourth column; mean fraction rightward choices ± binomial CIs, psychometric curves/RTs conditioned on previous right wins (blue) or left wins (pink)). Data represented by points (circles: choices, squares: reaction times) and model fits represented by lines (choices) or shaded bars (reaction times, thickness represents 95% bootstrap prediction intervals). n = 33462 trials (K294), 40350 trials (K299), 52471 trials (X005), 19055 trials (X017), 24413 trials (X018), and 53480 trials (X005)

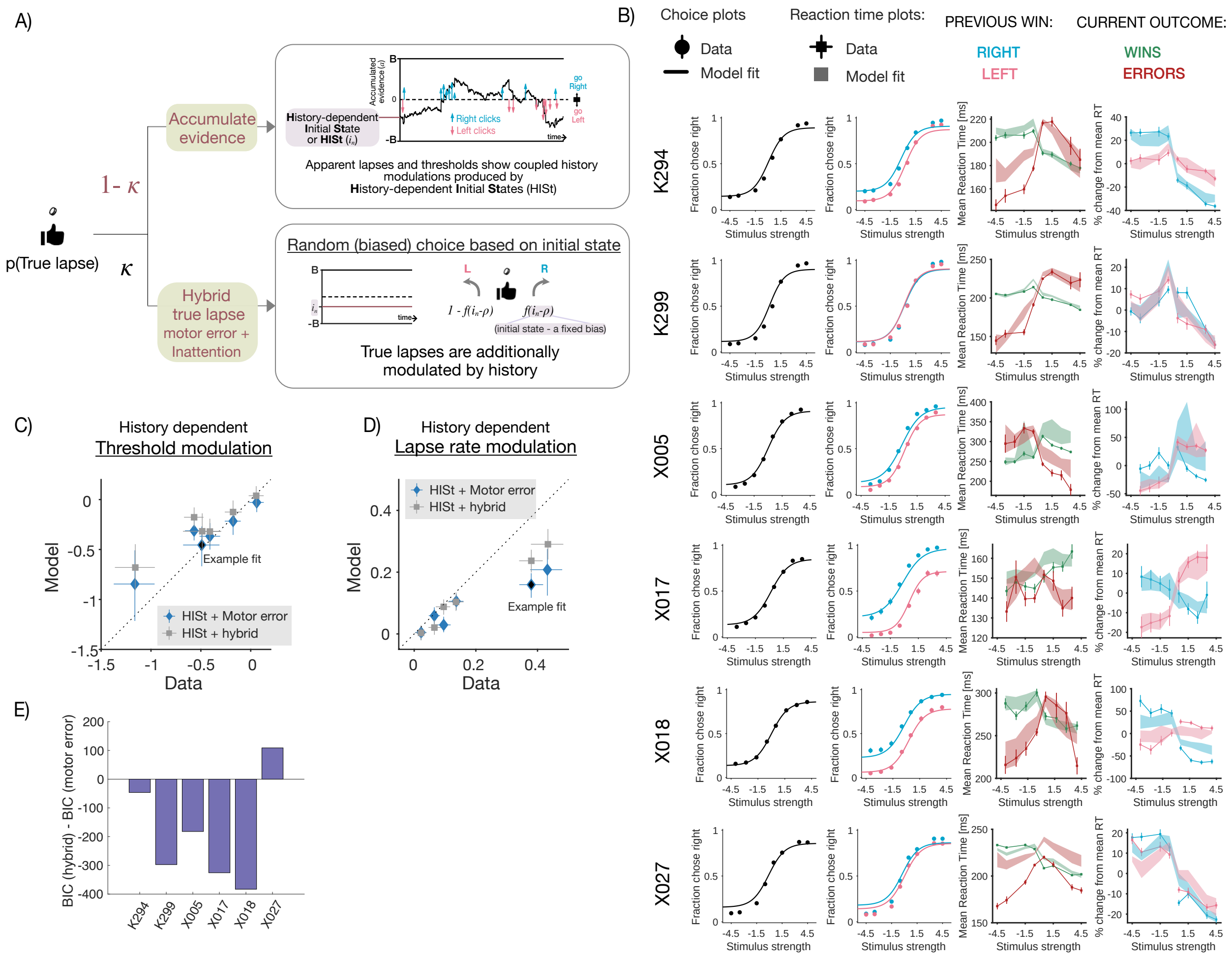

Supplementary Figure 9: **Accumulator model with HISt and true lapse variants fit to the RT dataset** **(A)** Flexible true lapse variant of the accumulator model with HISt, capable of producing both motor errors and inattentional true lapses: in this model, the subject chooses stochastically on true lapse trials, with a probability given by a sigmoidal function of the initial state (such a strategy is often called probability matching and although suboptimal, has found empirical support in many perceptual tasks e.g. Mamassian et al.[6]). The slope parameter of the sigmoid which is fit to the data – when this slope parameter goes to infinity, this model picks deterministically based on the initial state, similar to the previous inattention model. On the other hand when the slope goes to zero, choices on true lapse trials are no longer dependent on initial states, reducing to the motor error/exploration model, with intermediate parameters interpolating between these two extremes. This "hybrid true lapse" variant of the model can flexibly include many kinds of true lapses (Figure adapted with permission from Bingni W. Brunton et al., Rats and Humans Can Optimally Accumulate Evidence for Decision Making. Science 340,95-98(2013). DOI:10.1126/science.1233912) **(B)** Fits of the hybrid model to individual rats in the RT dataset, showing choice, RT and history measures similar to Supp Fig. 8. **C-D** History modulations in psychometric thresholds (C) and lapse rates (D) for motor error (blue) and hybrid (grey) models. Once again, allowing for the possibility of history-modulated true lapses slightly improves correspondence to lapse rate modulations, at the cost of threshold modulations. **(E)** Difference in BIC scores between the hybrid and motor error models, for individual rats in the reaction time dataset. Negative values indicate that the more flexible hybrid model won, as is the case for most rats. n = 33462 trials (K294), 40350 trials (K299), 52471 trials (X005), 19055 trials (X017), 24413 trials (X018), and 53480 trials (X005)

References

[1] Yu, A. & Cohen, J. Sequential effects: Superstition or rational behavior?. *Advances In Neural Information Processing Systems*. **21** (2008)

[2] Schütt, H., Harmeling, S., Macke, J. & Wichmann, F. Painfree and accurate Bayesian estimation of psychometric functions for (potentially) overdispersed data. *Vision Research*. **122** pp. 105-123 (2016)

[3] Wang, M., Montanède, C., Chandrasekaran, C., Peixoto, D., Shenoy, K. & Kalaska, J. Macaque dorsal premotor cortex exhibits decision-related activity only when specific stimulus–response associations are known. *Nature Communications*. **10**, 1793 (2019)

[4] Hermoso-Mendizabal, A., Hyafil, A., Rueda-Orozco, P., Jaramillo, S., Robbe, D. & La Rocha, J. Response outcomes gate the impact of expectations on perceptual decisions. *Nature Communications*. **11**, 1057 (2020)

[5] Karlsson, M., Tervo, D. & Karpova, A. Network resets in medial prefrontal cortex mark the onset of behavioral uncertainty. *Science*. **338**, 135-139 (2012)

[6] Mamassian, P., Landy, M. & Maloney, L. Bayesian modelling of visual perception. *Probabilistic Models Of The Brain: Perception And Neural Function*. pp. 13-36 (2002)
