# Supplementary material for: Trial-history biases in evidence accumulation can give rise to apparent lapses in decision-making
Source: Nat Commun. 2024 Jan 22;15:662. doi: 10.1038/s41467-024-44880-5 (PMC10803295; doi:10.1038/s41467-024-44880-5)
Supplement: Supplementary file 3 — Reporting Summary [file 41467_2024_44880_MOESM3_ESM.pdf]

Corresponding author(s): Dr. Carlos Brody

Last updated by author(s): Dec 5, 2023

Reporting Summary

Nature Portfolio wishes to improve the reproducibility of the work that we publish. This form provides structure for consistency and transparency in reporting. For further information on Nature Portfolio policies, see our Editorial Policies and the Editorial Policy Checklist.

Statistics

For all statistical analyses, confirm that the following items are present in the figure legend, table legend, main text, or Methods section.

n/a	Confirmed	
☐	☒	The exact sample size (*n*) for each experimental group/condition, given as a discrete number and unit of measurement
☐	☒	A statement on whether measurements were taken from distinct samples or whether the same sample was measured repeatedly
☐	☒	The statistical test(s) used AND whether they are one- or two-sided<br>*Only common tests should be described solely by name; describe more complex techniques in the Methods section.*
☒	☐	A description of all covariates tested
☐	☒	A description of any assumptions or corrections, such as tests of normality and adjustment for multiple comparisons
☐	☒	A full description of the statistical parameters including central tendency (e.g. means) or other basic estimates (e.g. regression coefficient) AND variation (e.g. standard deviation) or associated estimates of uncertainty (e.g. confidence intervals)
☐	☒	For null hypothesis testing, the test statistic (e.g. *F*, *t*, *r*) with confidence intervals, effect sizes, degrees of freedom and *P* value noted<br>*Give P values as exact values whenever suitable.*
☒	☐	For Bayesian analysis, information on the choice of priors and Markov chain Monte Carlo settings
☒	☐	For hierarchical and complex designs, identification of the appropriate level for tests and full reporting of outcomes
☐	☒	Estimates of effect sizes (e.g. Cohen's *d*, Pearson's *r*), indicating how they were calculated

Our web collection on statistics for biologists contains articles on many of the points above.

Software and code

Policy information about availability of computer code

Data collection	Data collection was performed using custom MATLAB code based on a previously published training protocol (Brunton 2013). The training code for both protocols are version controlled and maintained on github in a private repository, but can be made available upon reasonable request via email to the corresponding author.
Data analysis	Data analysis was performed using custom MATLAB and Julia code that have been made available on a github repository: https://github.com/Brody-Lab/trialhistory_lapses_EA.git with DOI: 10.5281/zenodo.10161051

For manuscripts utilizing custom algorithms or software that are central to the research but not yet described in published literature, software must be made available to editors and reviewers. We strongly encourage code deposition in a community repository (e.g. GitHub). See the Nature Portfolio guidelines for submitting code & software for further information.

Data

Policy information about availability of data

All manuscripts must include a data availability statement. This statement should provide the following information, where applicable:

- Accession codes, unique identifiers, or web links for publicly available datasets
- A description of any restrictions on data availability
- For clinical datasets or third party data, please ensure that the statement adheres to our policy

The rodent behavioral data generated in this study from the Poisson Clicks and the reaction time task have been deposited in the figshare database under accession code CC BY 4.0 at the following DOI: 10.6084/m9.figshare.24113793. Source data are provided with this paper.

Research involving human participants, their data, or biological material

Policy information about studies with human participants or human data. See also policy information about sex, gender (identity/presentation), and sexual orientation and race, ethnicity and racism.

Reporting on sex and gender	N/A
Reporting on race, ethnicity, or other socially relevant groupings	N/A
Population characteristics	N/A
Recruitment	N/A
Ethics oversight	N/A

Note that full information on the approval of the study protocol must also be provided in the manuscript.

Field-specific reporting

Please select the one below that is the best fit for your research. If you are not sure, read the appropriate sections before making your selection.

☒ Life sciences ☐ Behavioural & social sciences ☐ Ecological, evolutionary & environmental sciences

For a reference copy of the document with all sections, see nature.com/documents/nr-reporting-summary-flat.pdf

Life sciences study design

All studies must disclose on these points even when the disclosure is negative.

Sample size	No explicit sample size calculation was performed. Sample sizes (subject numbers, trials per subject) were determined by the limits of data collection within a reasonable timeframe and precedent in the literature.
Data exclusions	For the data from Poisson clicks task, we analyzed rats which performed greater than 30,000 trials, at 70% or more accuracy. Sessions with less than 300 trials or less than 60% accuracy for either of the choices were excluded. Since rats typically perform this task for many months after having passed the final training stage, to minimize nonstationarities in the data (due to break in training because of holiday closures etc.) and ensure that we are analyzing asymptotic performance, we identified temporally contiguous sessions with stable accuracy by performing change-point detection on smoothed trial hit rate using MATLAB's findchangepts function. The partition with most number of trials was included in the analysis.<br><br>For the data from reaction time version of the Poisson Clicks task, rats whose performance did not meet the criterion of 70% accuracy even after a week of training with reaction time manipulation were excluded. Rats tended to have worse accuracy early in the session, so we omitted the first 50 trials from our analysis. After the first 50 trials, we confirmed that the accuracy in the first and second halves of the session was comparable.
Replication	We verify the results across the two datasets analyzed in this manuscript (152 Poisson clicks rats, and 6 reaction time rats). Statistical methods were used to ensure that the findings are significant and are able to be replicated.
Randomization	No group randomization was required or performed for this study. The order of trial types presented to each subject was randomized.
Blinding	Our study did not require comparisons between groups, hence blinding was deemed unnecessary.

Reporting for specific materials, systems and methods

We require information from authors about some types of materials, experimental systems and methods used in many studies. Here, indicate whether each material, system or method listed is relevant to your study. If you are not sure if a list item applies to your research, read the appropriate section before selecting a response.

Materials & experimental systems

n/a	Involved in the study	
☒	☐	Antibodies
☒	☐	Eukaryotic cell lines
☒	☐	Palaeontology and archaeology
☐	☒	Animals and other organisms
☒	☐	Clinical data
☒	☐	Dual use research of concern
☒	☐	Plants

Methods

n/a	Involved in the study	
☒	☐	ChIP-seq
☒	☐	Flow cytometry
☒	☐	MRI-based neuroimaging

Animals and other research organisms

Policy information about studies involving animals; ARRIVE guidelines recommended for reporting animal research, and Sex and Gender in Research

Laboratory animals	Male Long-Evans rats (Rattus norvegicus), aged 6-26 months were used for this study.
Wild animals	The study did not involve wild animals.
Reporting on sex	Sex was not considered in the study design, all animals used in the study were males. Housing both male and female rats in our rodent system led to very substantially increased aggressiveness, particularly among some of our transgenic rat lines, to the point of making these rats unsafe to handle and preventing us from studying both sexes. A major reorganization of the housing will allow us to avoid this problem going forward, but at the time of this study this new system was not yet in place, and therefore the data was gathered from male rats only.
Field-collected samples	The study did not involve field-collected samples.
Ethics oversight	Animal use procedures were approved by the Princeton University Institutional Animal Care and Use Committee (IACUC #1853).

Note that full information on the approval of the study protocol must also be provided in the manuscript.
